# Supplementary material for: Integrated proteogenomic characterization of localized prostate cancer identifies biological insights and subtype-specific therapeutic strategies
Source: Nat Commun. 2025 Apr 3;16:3189. doi: 10.1038/s41467-025-58569-w (PMC11968977; doi:10.1038/s41467-025-58569-w)
Supplement: Supplementary file 1 — Supplementary information [file 41467_2025_58569_MOESM1_ESM.docx]

**Supplementary Information**

**Integrated Proteogenomic Characterization of Localized Prostate Cancer Identifies Biological Insights and Subtype-Specific Therapeutic Strategies**

Wei Ou *et al.*

*Corresponding author: Zong-Ren Wang, wangzr27@mail.sysu.edu.cn; Ling-Li Long, longll@mail.sysu.edu.cn; Ling-Wu Chen, chenlwu@mail.sysu.edu.cn.

# Supplementary Table

**Supplementary Table 1. Clinical characteristics of the discovery cohort.**

|  | The discovory cohort (n=145) |
| --- | --- |
| Age, years |  |
| ≤65 | 48 (33.1%) |
| 66-70 | 47 (32.4%) |
| 71-75 | 28 (19.3%) |
| >75 | 22 (15.2%) |
| PSA^a^, ng/mL |  |
| <10 | 32 (22.1%) |
| 10-20 | 46 (31.7%) |
| >20 | 66 (45.5%) |
| Risk group |  |
| Low | 8 (5.5%) |
| Intermediate | 36 (24.8%) |
| High | 101 (69.6%) |
| Gleason score |  |
| 6 | 14 (9.6%) |
| 7 | 70 (48.3%) |
| ­ 8 | 29 (20.0%) |
| 9 | 29 (20.0%) |
| 10 | 2 (1.4%) |
| Postoperative T stage |  |
| pT2 | 92 (63.4%) |
| pT3a | 19 (13.1%) |
| pT3b | 34 (23.4%) |
| Postoperative N stage |  |
| pN0 | 131 (90.3%) |
| pN1 | 14 (9.6%) |
| BCR^b^, n (%) |  |
| Yes | 41 (28.3%) |
| No | 102 (70.3%) |
| Not available | 2 (1.4%) |
| ^a^PSA = prostate-specific antigen  ^b^BCR = biochemical recurrence | |

**Supplementary Table 2. The multivariable cox regression for the biochemical recurrence-free survival (including proteomic subgroups) in the discovery cohort.**

| Variables | HR | 95%CI | *P-values |
| --- | --- | --- | --- |
| Age (years) | 0.96 | (0.92,1.01) | 0.132 |
| PSA (10ng/ml) | 1.03 | (1.00,1.05) | 0.027 |
| Gleason score |  |  |  |
| <=7 | 1.00 |  |  |
| >=8 | 2.35 | (1.11,5.01) | 0.026 |
| T-stage |  |  |  |
| pT2 | 1.00 |  |  |
| pT3 | 1.63 | (0.81,3.30) | 0.172 |
| N-stage |  |  |  |
| pN0 | 1.00 |  |  |
| pN1 | 1.25 | (0.52,3.03) | 0.621 |
| Proteomic subgroups |  |  |  |
| Subgroup3 | 1.00 |  |  |
| Subgroup2 | 2.53 | (1.15,5.58) | 0.021 |
| Subgroup1 | 1.91 | (0.75,4.88) | 0.174 |

*P-values are determined using the two-sided Wald test and FDR correction.

**Supplementary Table 3. The multivariable cox regression for the biochemical recurrence-free survival (including NANS expression level) in the discovery cohort.**

| Variables | HR | 95%CI | *P-values |
| --- | --- | --- | --- |
| TMB | 1.12 | (0.60,2.09) | 0.715 |
| SCNA burden | 0.99 | (0.93,1.05) | 0.687 |
| Age(years) | 0.98 | (0.93,1.03) | 0.354 |
| PSA (10ng/ml) | 1.03 | (1.00,1.05) | 0.042 |
| Gleason score |  |  |  |
| <=7 | 1.00 |  |  |
| >=8 | 2.49 | (1.17,5.30) | 0.018 |
| T-stage |  |  |  |
| pT2 | 1.00 |  |  |
| pT3 | 1.64 | (0.79,3.42) | 0.184 |
| N-stage |  |  |  |
| pN0 | 1.00 |  |  |
| pN1 | 1.38 | (0.56,3.41) | 0.487 |
| NANS group |  |  |  |
| NANS low | 1.00 |  |  |
| NANS high | 2.51 | (1.06,5.93) | 0.035 |

*P-values are determined using the two-sided Wald test and FDR correction.

**Supplementary Table 4. Detailed information of cell lines and antibodies.**

| **Reagent or Resource** | **Source** | **Identifier** |
| --- | --- | --- |
| **Cell lines** | | |
| RM1 | iCell | iCell-m051 |
| Myc-CaP | CTCC | CTCC-001-0359 |
| Vcap | iCell | iCell-h222 |
| 22Rv1 | procell | CL-0004 |
| DU145 | iCell | iCell-h250 |
| PC3 | iCell | iCell-h174 |
| C4-2 | BNCC | BNCC338093-2 |
| C4-2B | BNCC | BNCC341733-2 |
| **Antibodies** | | |
| Rabbit polyclonal anti-NANS | Novus | NBP1-87088 |
| Rabbit monoclonal anti-CD4 | abcam | ab133616 |
| Mouse monoclonal anti-CD8a | CST | 70306S |
| Mouse monoclonal anti-CD20 | abcam | ab9475 |
| Rabbit monoclonal anti-CD163 | abcam | ab182422 |
| Sambucus Nigra Lectin (SNA, EBL), Biotinylated | Vector | B-1305-2 |
| Maackia Amurensis Lectin II (MAL II), Biotinylated | Vector | B-1265-1 |

**Supplementary Table 5. Culture condition of cell lines.**

| **Cell line** | **Media** | **Serum** |
| --- | --- | --- |
| RM1 | DMEM | FBS |
| Myc-CaP | DMEM | FBS |
| Vcap | DMEM | FBS |
| 22Rv1 | 1640 | FBS |
| DU145 | DMEM | FBS |
| PC3 | 1640 | FBS |
| C4-2 | 1640 | FBS |
| C4-2B | 1640 | FBS |

# Supplementary Figure


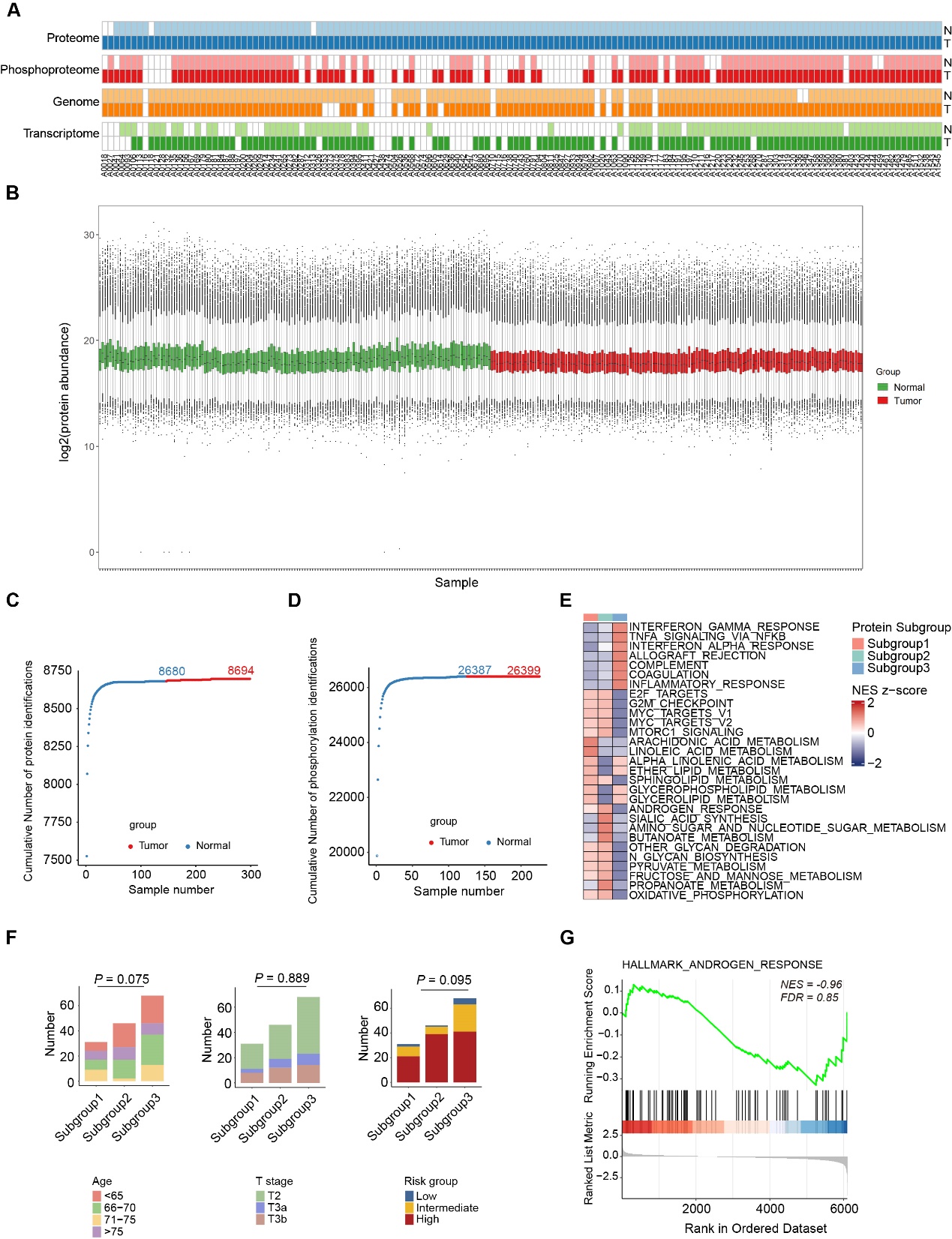


**Supplementary Figure 1. Quality assessments for multi-omics data**

1. Sample distributions of multi-omics analysis, including genome, transcriptome, proteome and phosphoproteome.
2. Distribution of log2-transformed protein abundance of identified proteins in 286 proteome samples that passed quality control. Green presents non-tumor samples (n =141), red denotes tumor samples (n = 145). In the box plots, the middle bar represents the median, and the box represents the IQR; bars extend to 1.5 times IQR.
3. Cumulative number of protein identifications in tumor and non-tumor samples. Blue presents non-tumour samples (n =141), red denotes tumour samples (n = 145).
4. Cumulative number of phosphorylation site identifications in tumor and non-tumor samples. Blue presents non-tumor samples (n =87), red denotes tumor samples (n = 107).
5. Gene sets enrichment analysis (GSEA) showing distinct transcriptomic molecular characteristics among three proteomic subgroups of the discovery cohort.
6. Bar plots comparing the clinicopathological features among three proteomic subgroups of the discovery cohort, including age, tumor stage and risk group. P-values are determined using one-way ANOVA test.
7. GSEA showing the down-regulation of the androgen response pathway in subgroup 1 compared to subgroup 2 and 3 combined of the discovery cohort.

Source data are provided as a Source Data file.


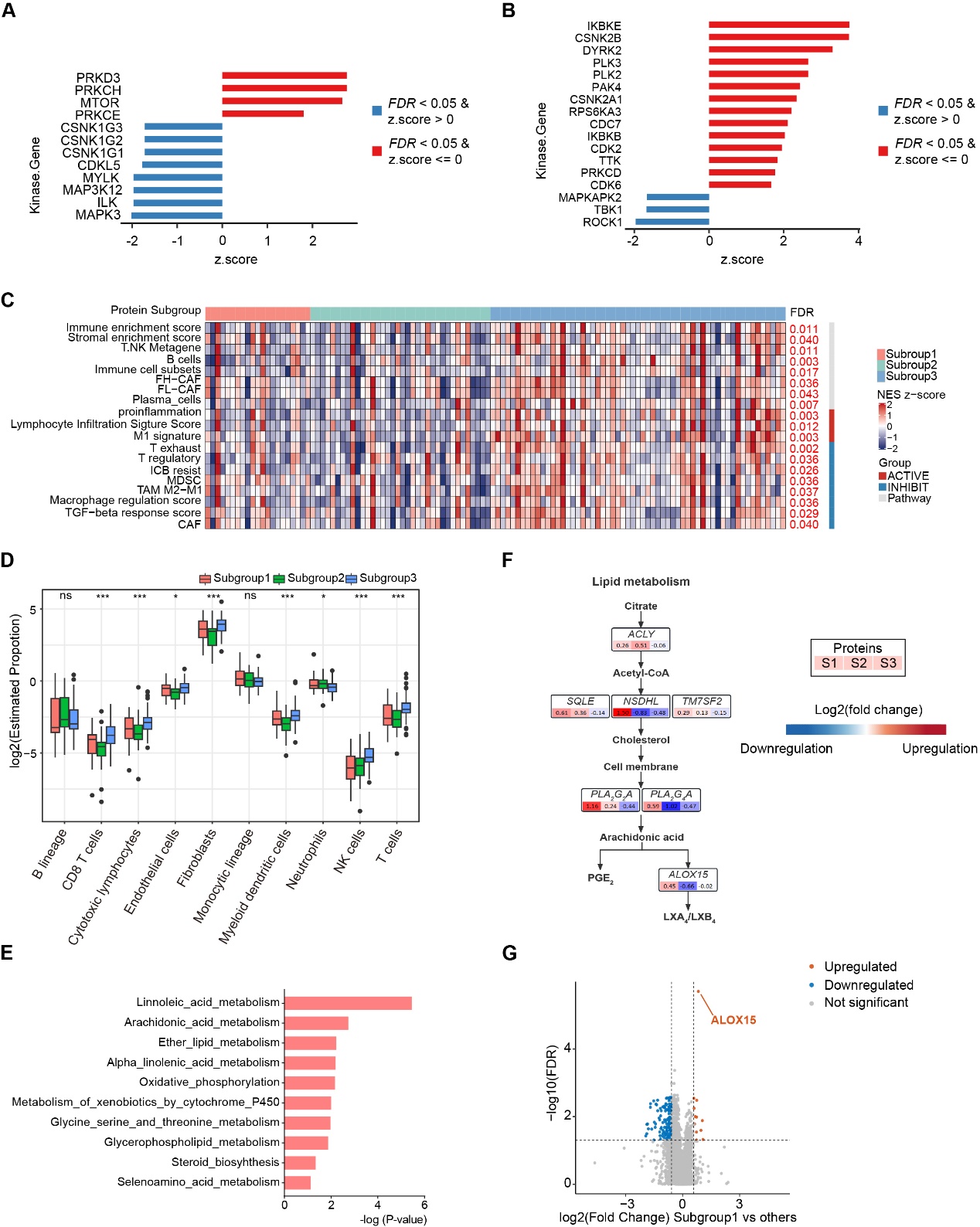


**Supplementary Figure 2. Molecular features of subgroup 1 and 3.**

1. Kinase-Substrate Enrichment Analysis (KSEA) showed significantly up-regulated and down-regulated kinases in subgroup 1. Red bars refer to FDR< 0.05 and z score > 0, blue bars refer to FDR< 0.05 and z score ≤ 0.
2. Kinase-Substrate Enrichment Analysis (KSEA) showed significantly up-regulated and down-regulated kinases in subgroup 3. Red bars refer to FDR< 0.05 and z score > 0, blue bars refer to FDR< 0.05 and z score ≤ 0.
3. Single-sample gene set enrichment analysis (ssGSEA) of immune-related pathways among three proteomic subgroups in the discovery cohort. FDR values are determined using Kruskal-Wallis test and Benjamini-Hochberg (B-H) correction.
4. MCP-counter analysis showing immune cell infiltrations among three proteomic subgroups in the discovery cohort. P-values are determined using Kruskal-Wallis test and Benjamini-Hochberg (B-H) correction.(N.S. p>0.05; *p<0.05 and ***p<0.001).
5. GSEA showing the top 10 up-regulated metabolic pathways in subgroup 1 patients of the discovery cohort (subgroup 1 vs subgroup 2 and subgroup 3 combined).
6. Differential expression analysis of key enzymes involved in the arachidonic acid metabolism pathway among three proteomic subgroups in the discovery cohort.
7. Volcano plot showing higher expression of ALOX15 protien in subgroup 1 compared to subgroup 2 and 3 of the discovery cohort. FDR values are determined using two-sided Wilcoxon signed-rank test and B-H correction.

Source data are provided as a Source Data file.


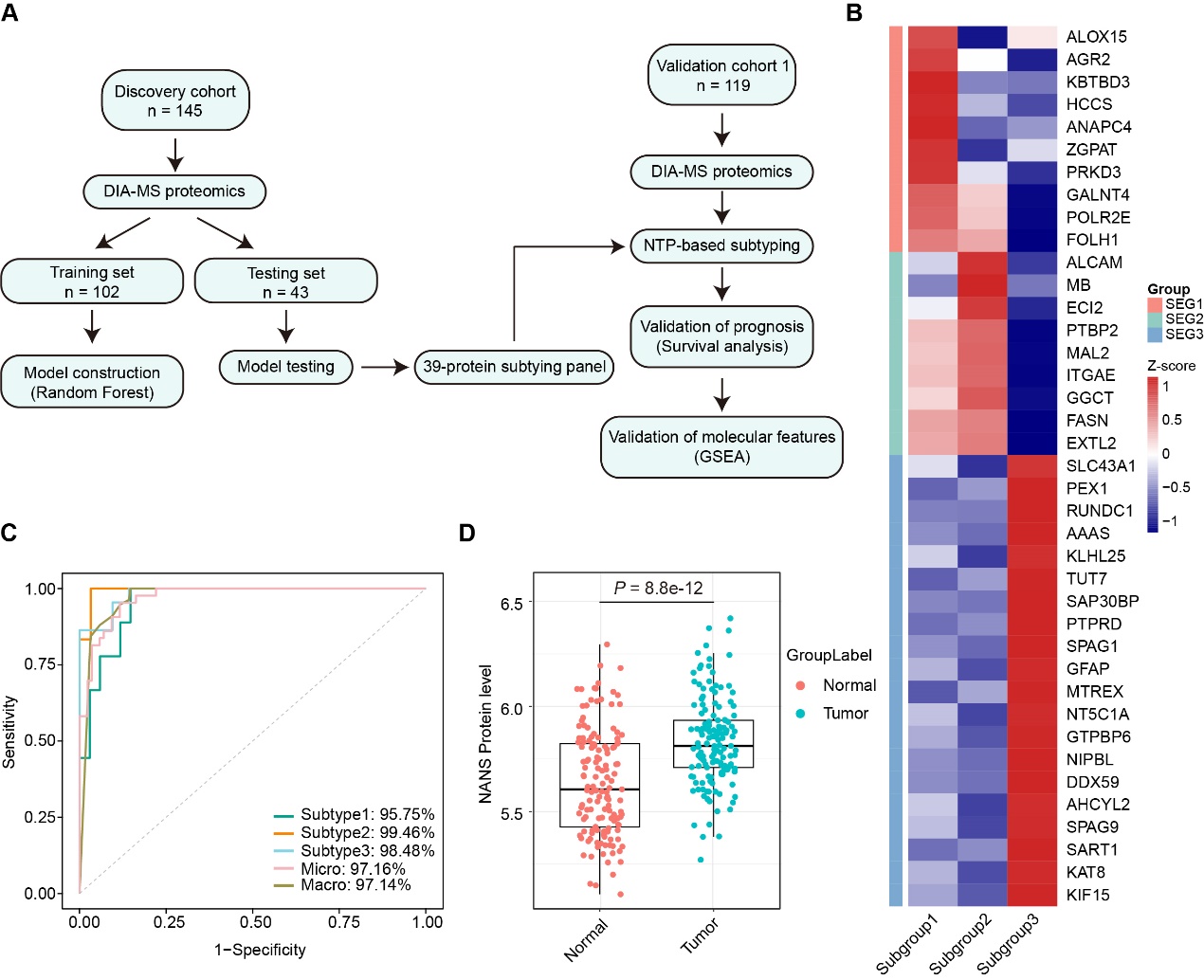


**Supplementary Figure 3. The 39-protein panel for proteomic subtyping of PCa and differential expression of NANS in tumors and paired normal tissues..**

1. Design of the analytical pipeline for construction and validation of the 39-protein subtyping panel.
2. The 39-protein panel for proteomic subtyping of PCa filtered by the Random Forest algorithm, SEG represents subgroup enriched genes.
3. The Receiver Operating Characteristic Curves (ROC) of the 39-protein subtyping model in the validation dataset.
4. Comparison of NANS protein expression between tumor and paired normal tissues in all patients of the discovery cohort. P-value is determined using the two-sided Wilcoxon rank-sum test.

Source data are provided as a Source Data file.


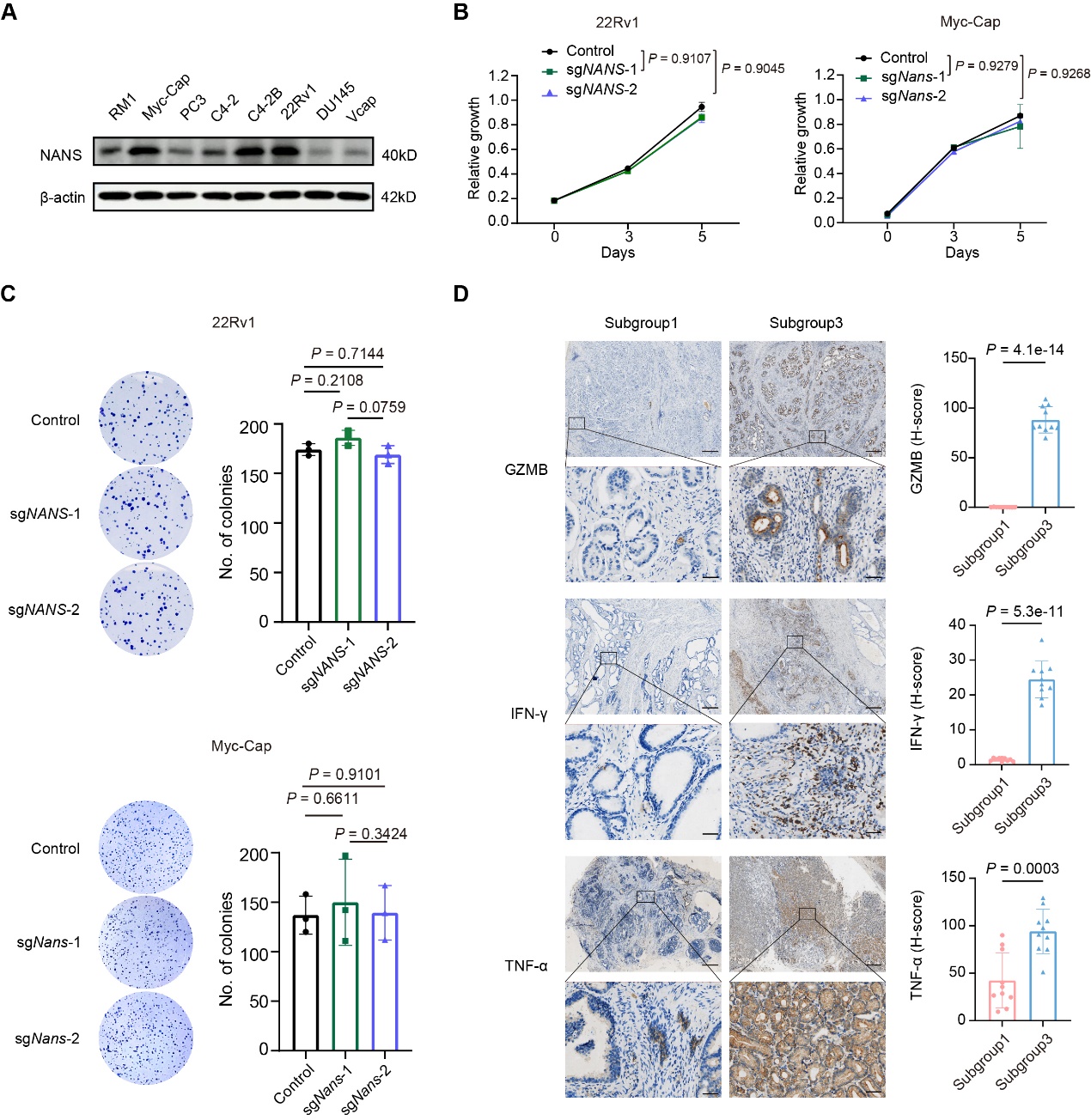


**Supplementary Figure 4. The role of NANS in regulating the proliferation of PCa cells.**

1. Comparison of NANS protein expression among 8 PCa cell lines by Western blot.
2. CCK8 assays for 22Rv1 and Myc-CaP cells of sg*NANS* and control group. P-values are determined using two-way ANOVA test.
3. Colony-forming assays for 22Rv1 and Myc-CaP cells of sg*NANS* and control group. Data are presented as mean ± Standard deviation (SD). P-values are determined using two-tailed Student’s t test.
4. Immunohistochemistry (IHC) staining and quantification for GZMB, INF-γ and TNF-α in tumor regions of subgroup 1 and subgroup 3 patients. The scale bars represent 500/50um in low/high-power field. Data are presented as mean ± standard deviation (SD). P-values are determined using two-tailed Student’s t test.

Source data are provided as a Source Data file.


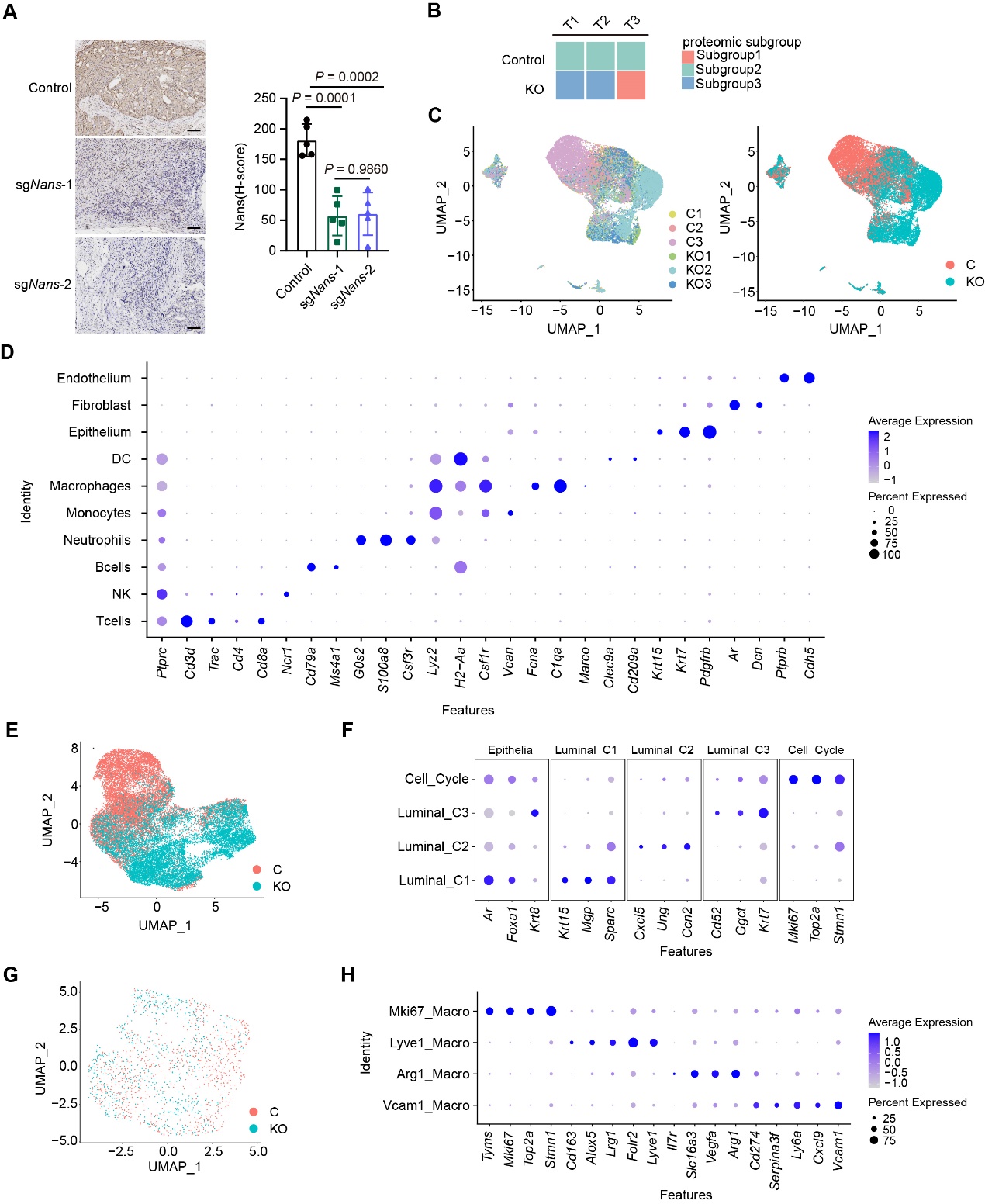


**Supplementary Figure 5. Inhibition of NANS reverses the immunosuppressive microenvironment of PCa in orthotopic transplanted mouse model.**

1. IHC staining and quantification for Nans in murine tumor tissues of the control and sg*Nans* group. n=5 for each group. The scale bar represents 100um. Data are presented as mean ± SD. P-values are determined using two-tailed Student’s t test.
2. UMAP visualization of 29515 single cells, (left panel) colored by sample origin (right panel) colored by group origin (control group or *Nans* KO group).
3. Marker gene expression for each cell type, where dot size and color represent percentage of marker gene expression (pct. exp) and the averaged scaled expression (avg. exp. scale) value, respectively.
4. UMAP visualization of epithelial cells, colored by group origin (control group or *Nans* KO group).
5. Marker gene expression for each epithelial cell type, where dot size and color represent percentage of marker gene expression (pct. exp) and the averaged scaled expression (avg. exp. scale) value, respectively.
6. UMAP visualization of macrophages, colored by group origin (control group or *Nans* KO group).
7. Marker gene expression for each macrophage cell type, where dot size and color represent percentage of marker gene expression (pct. exp) and the averaged scaled expression (avg. exp. scale) value, respectively.

Source data are provided as a Source Data file.


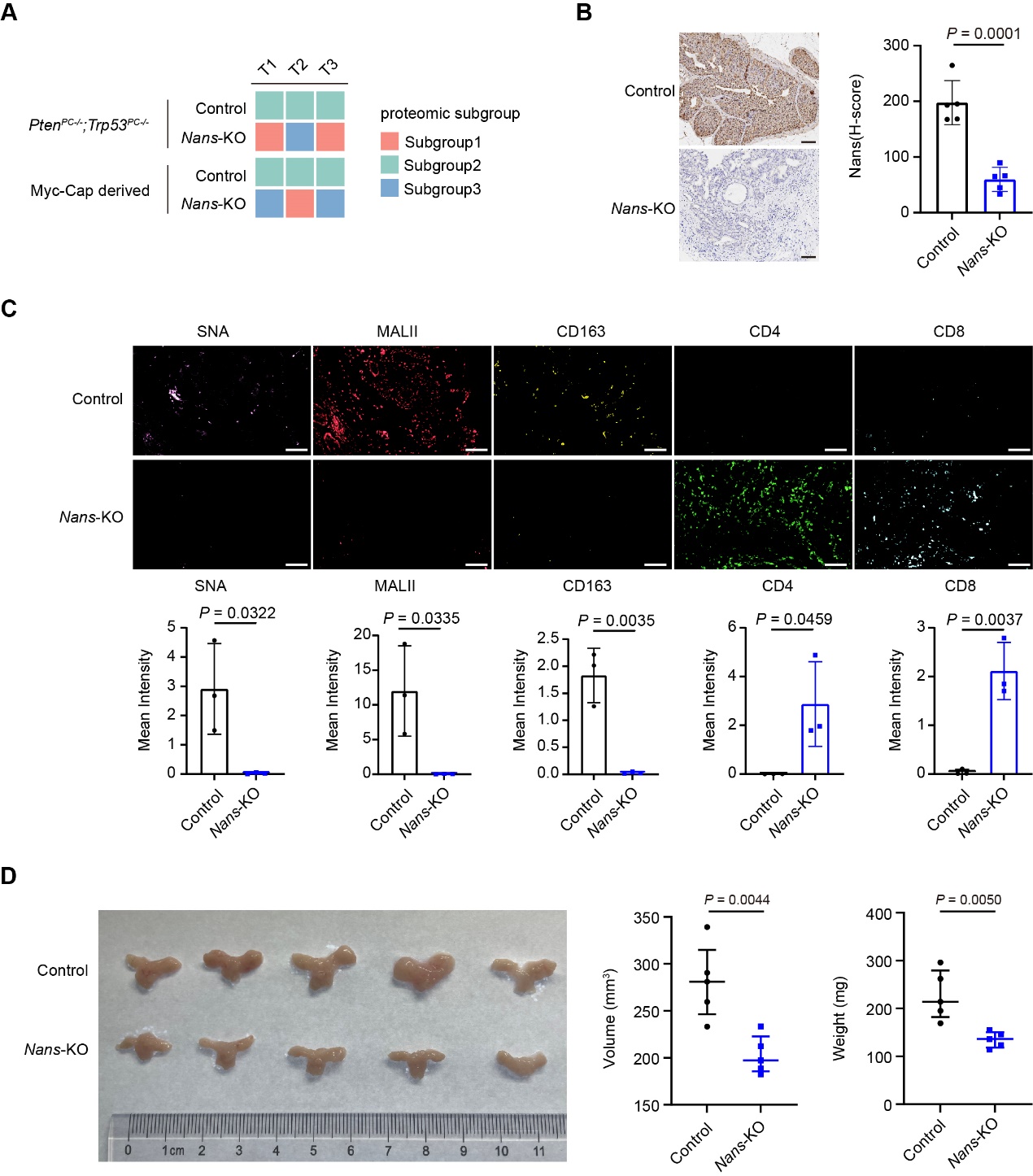


**Supplementary Figure 6. Targeting NANS suppresses the tumor growth of PCa in *Pten*^PC−/−^ ; *Trp53*^PC−/−^ mouse model.**

1. Heatmap visualizes the proteomic subtype assignments of murine tumor tissues of the control and *Nans* KO group in *Pten*^PC−/−^ ; *Trp53*^PC−/−^ and Myc-cap cell-derived mouse models. Three tumor samples for each group.
2. IHC staining and quantification for Nans in murine tumor tissues of the control and *Nans* KO group. n=5 for each group. The scale bar represents 100um. Data are presented as mean ± SD. P-values are determined using two-tailed Student’s t test.
3. IF staining and quantification of sialylation level (SNA/MALII) and immune cells in murine tumor tissues of the control and *Nans* KO group. n=3 for each group. The scale bar represents 50um. Data are presented as mean ± SD. P-values are determined using two-tailed Student’s t test.
4. Comparion of tumor volume and weights between the control and *Nans* KO group. n=5 for each group. P-values are determined using two-tailed Student’s t test.

Source data are provided as a Source Data file.
