# Supplementary figures and images for: Integrated proteogenomic characterization of localized prostate cancer identifies biological insights and subtype-specific therapeutic strategies
Source: Nat Commun. 2025 Apr 3;16:3189. doi: 10.1038/s41467-025-58569-w (PMC11968977; doi:10.1038/s41467-025-58569-w)

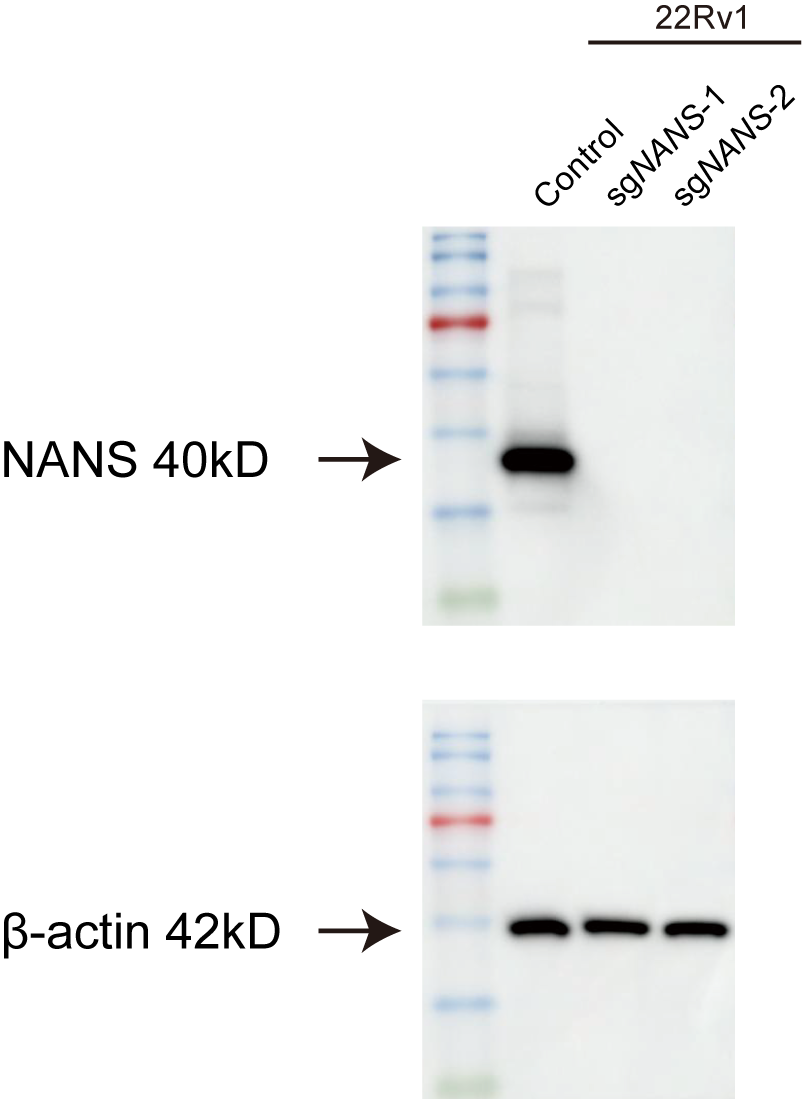

Supplement: Supplementary file 4 — Source data [file 41467_2025_58569_MOESM4_ESM.zip › SourceData/SourceData Figure 4/22RV1-Replicate 1.tif]

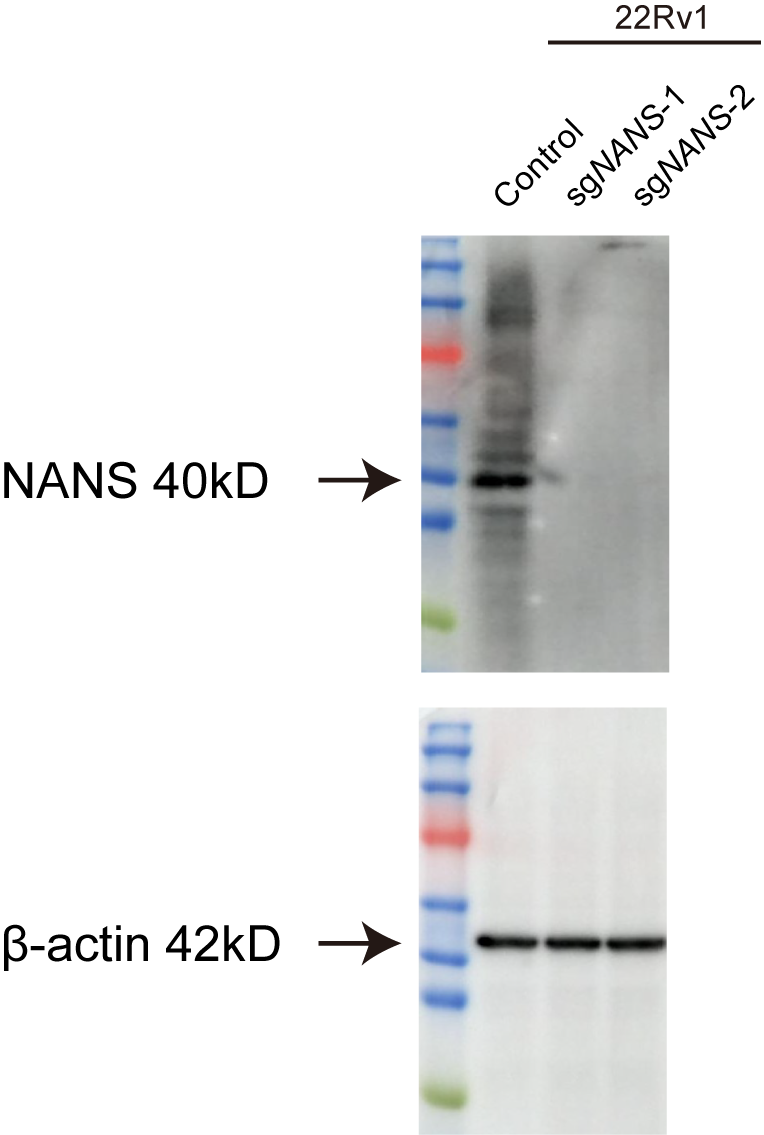

Supplement: Supplementary file 4 — Source data [file 41467_2025_58569_MOESM4_ESM.zip › SourceData/SourceData Figure 4/22RV1-Replicate 2.tif]

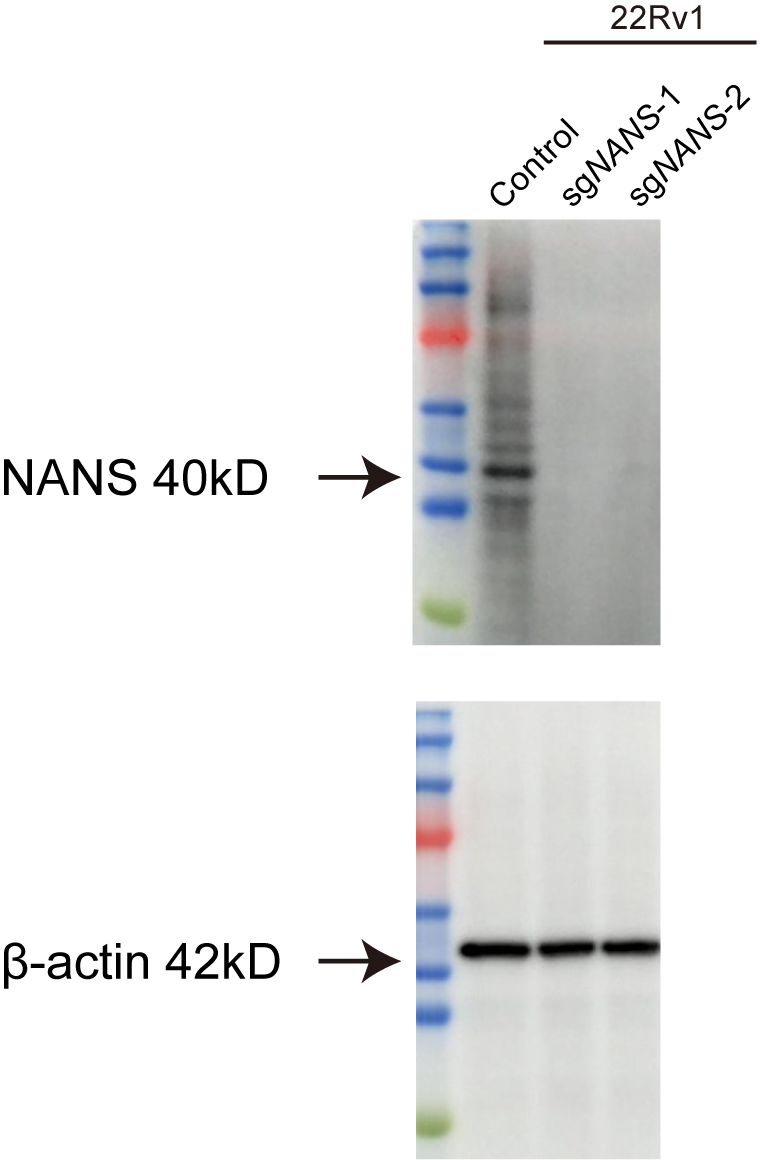

Supplement: Supplementary file 4 — Source data [file 41467_2025_58569_MOESM4_ESM.zip › SourceData/SourceData Figure 4/22RV1-Replicate 3.tif]

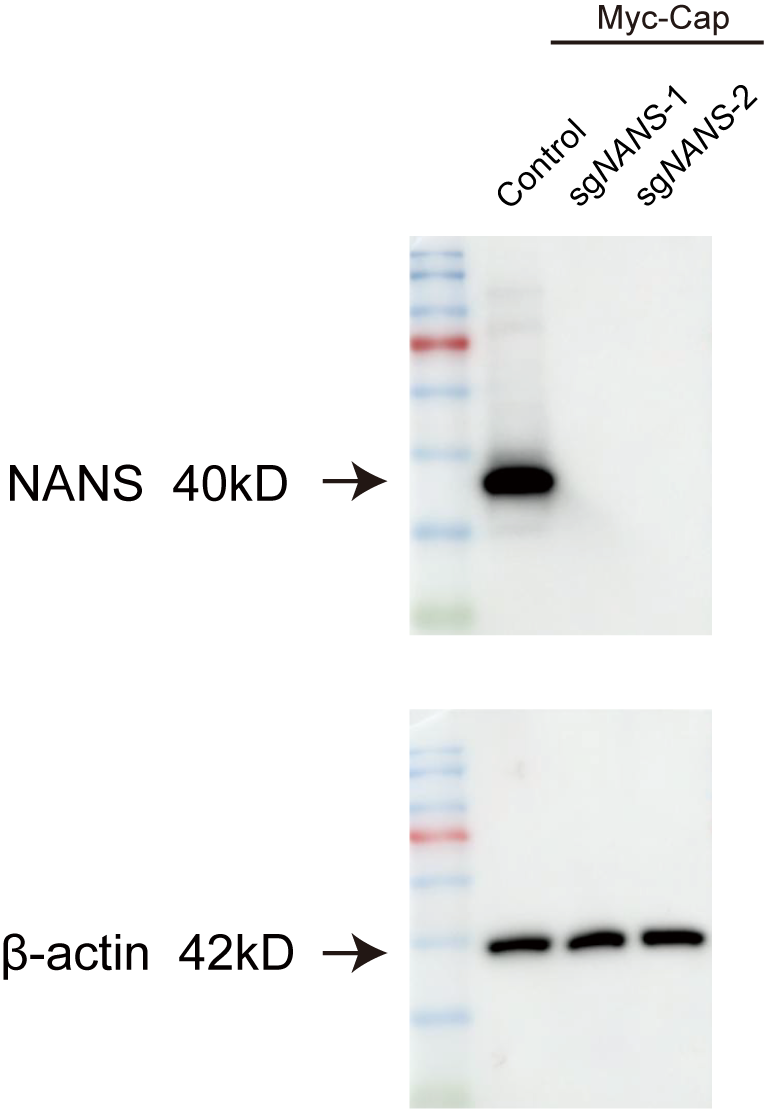

Supplement: Supplementary file 4 — Source data [file 41467_2025_58569_MOESM4_ESM.zip › SourceData/SourceData Figure 4/myccap-Replicate 1.tif]

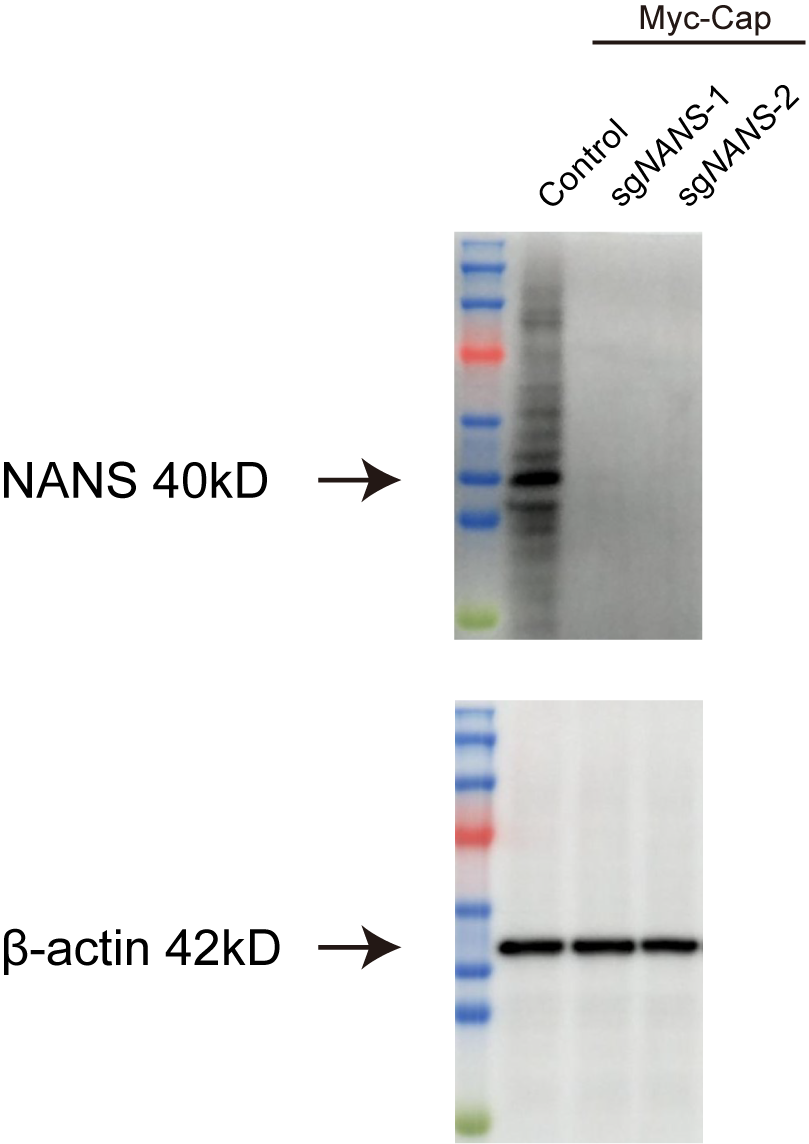

Supplement: Supplementary file 4 — Source data [file 41467_2025_58569_MOESM4_ESM.zip › SourceData/SourceData Figure 4/myccap-Replicate 2.tif]

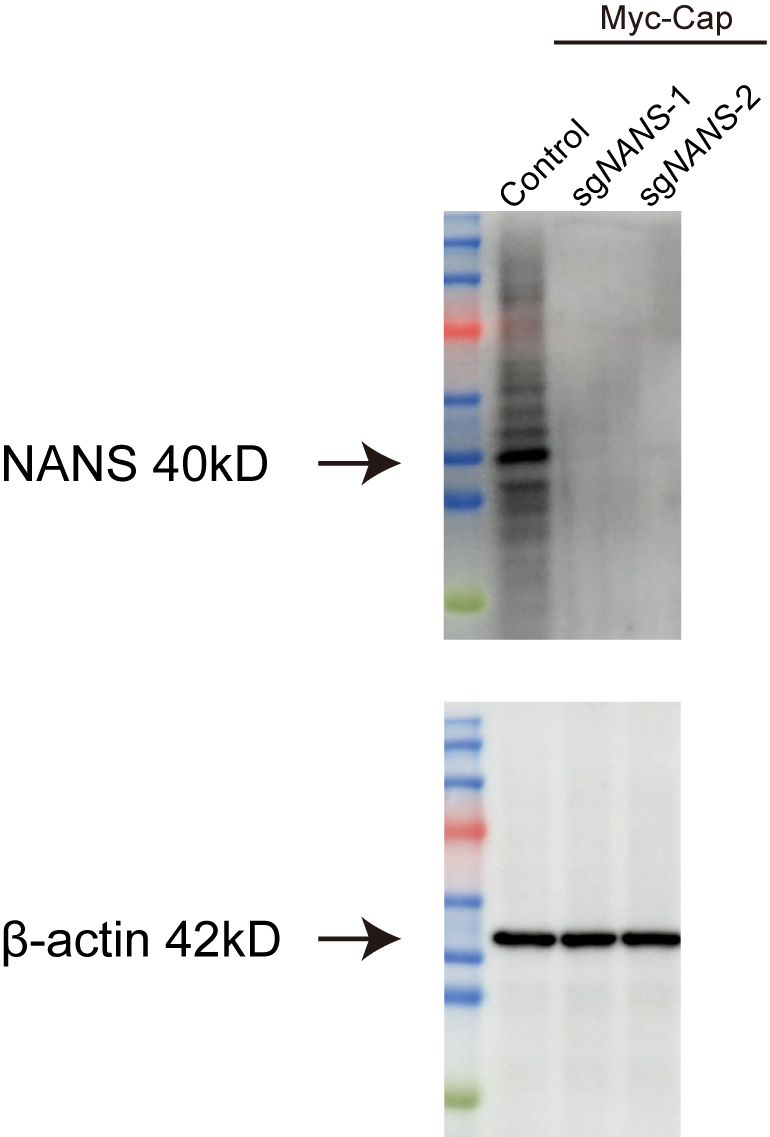

Supplement: Supplementary file 4 — Source data [file 41467_2025_58569_MOESM4_ESM.zip › SourceData/SourceData Figure 4/myccap-Replicate 3.tif]

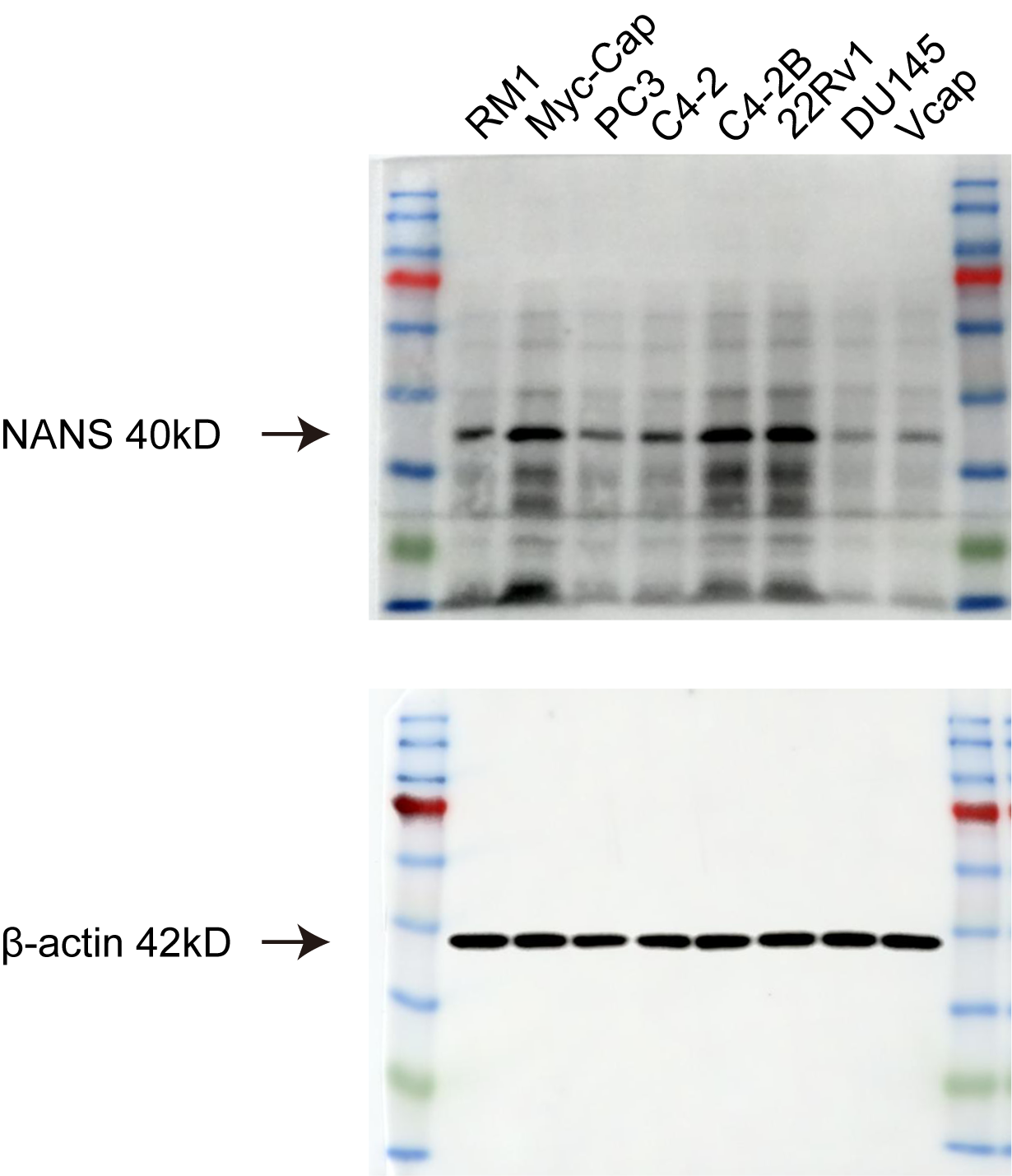

Supplement: Supplementary file 4 — Source data [file 41467_2025_58569_MOESM4_ESM.zip › SourceData/SourceData FigureS4/Replicate 1.tif]

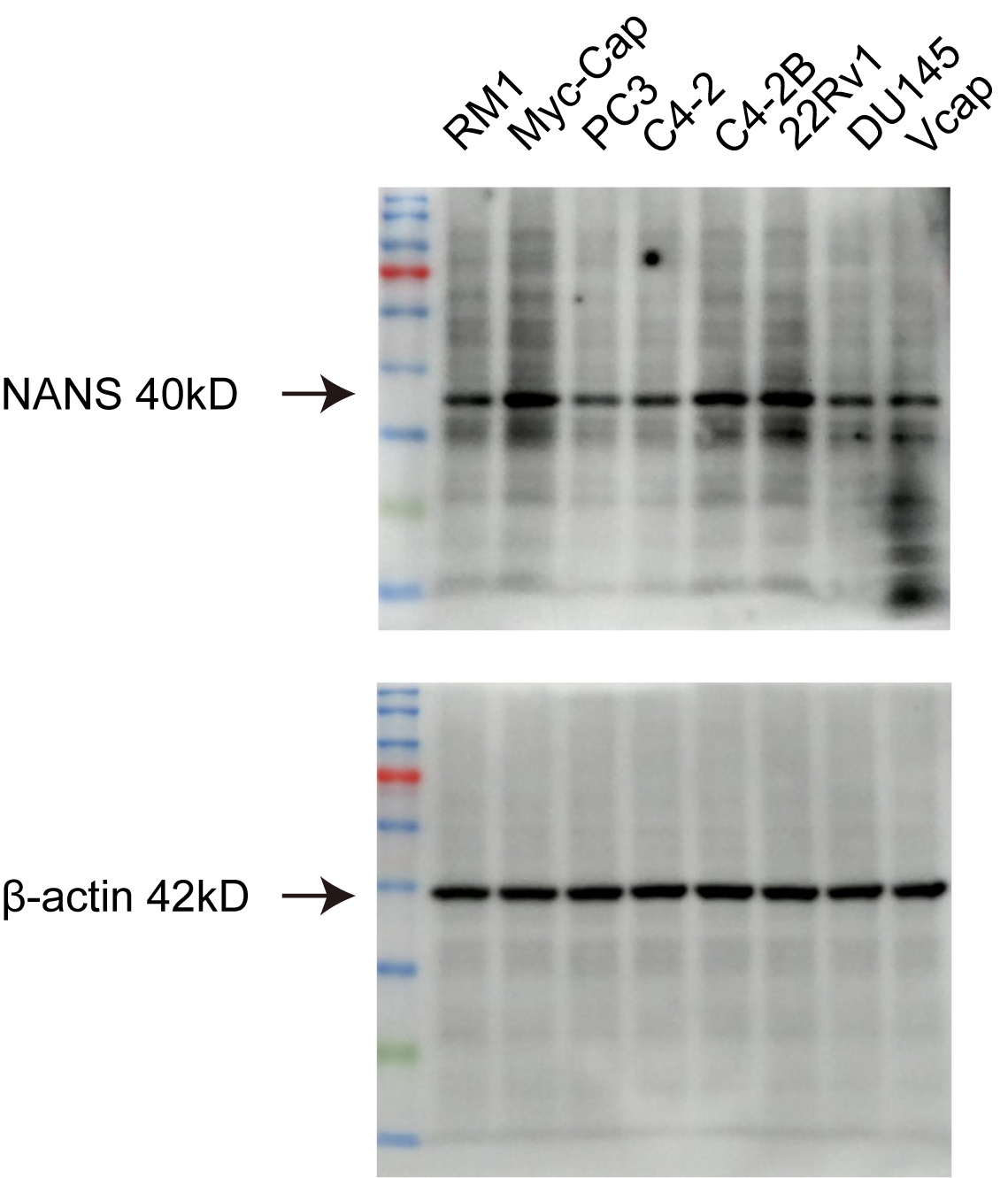

Supplement: Supplementary file 4 — Source data [file 41467_2025_58569_MOESM4_ESM.zip › SourceData/SourceData FigureS4/Replicate 2.tif]

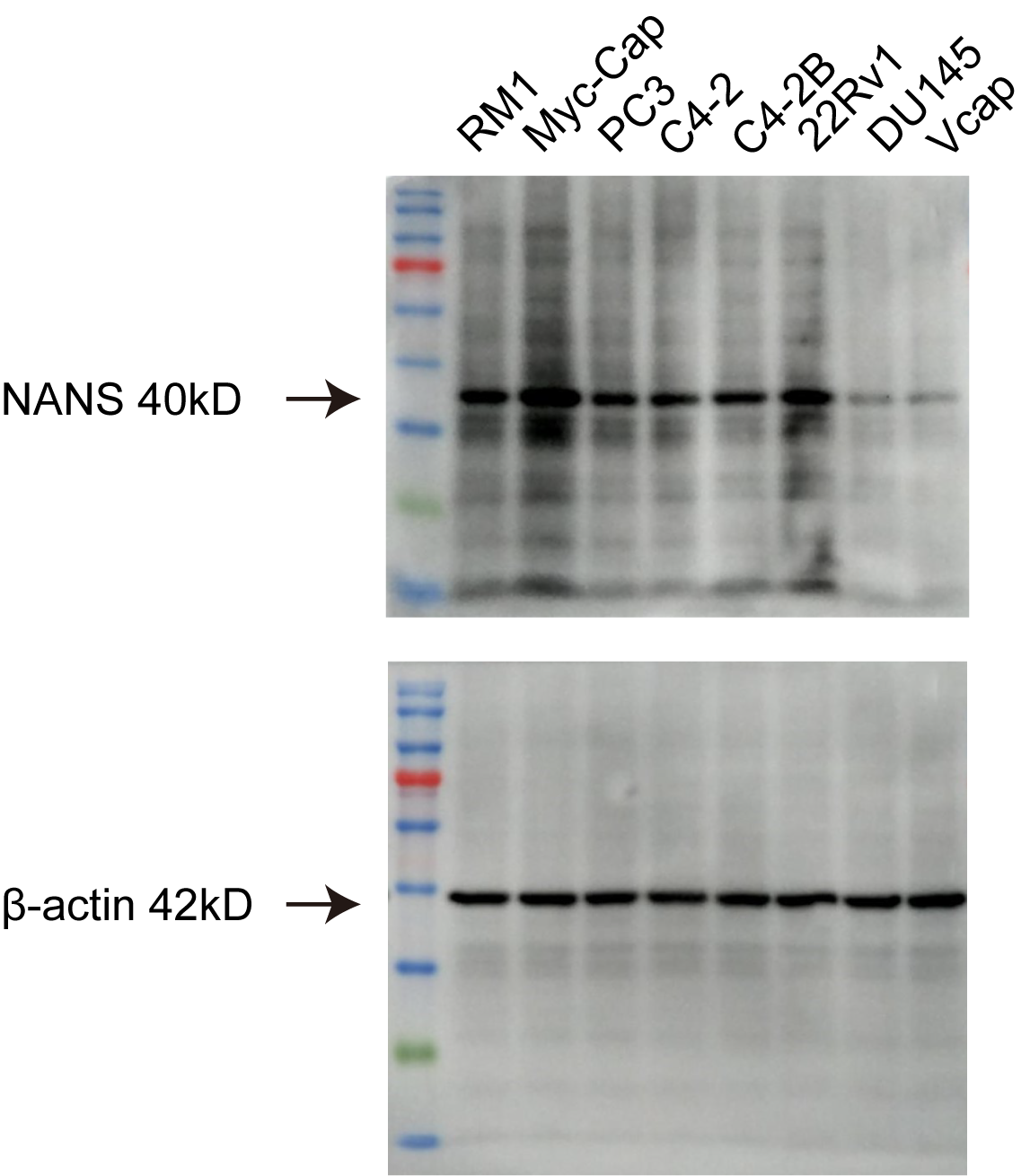

Supplement: Supplementary file 4 — Source data [file 41467_2025_58569_MOESM4_ESM.zip › SourceData/SourceData FigureS4/Replicate 3.tif]
